# Clec7a Signaling in Microglia Promotes Synapse Loss Associated with Tauopathy

**DOI:** 10.3390/ijms26072888

**Published:** 2025-03-22

**Authors:** Shubing Yang, Ji Wang, Yongkang Cao, Yibo Zhang, Zhuoran Sun, Pin Wan, Mingshan Pi, Qi Xiong, Xiji Shu, Xiaochuan Wang, Yiyuan Xia

**Affiliations:** 1 Hubei Key Laboratory of Cognitive and Affective Disorders, Jianghan University, Wuhan 430056, China; 2 Institutes of Biomedical Sciences, School of Medicine, Jianghan University, Wuhan 430056, China; 3 Department of Pathology and Pathophysiology, School of Medicine, Jianghan University, Wuhan 430056, China

**Keywords:** Alzheimer’s disease, tauopathy, Clec7a, MD2, synaptic degeneration

## Abstract

Alzheimer’s disease (AD) pathogenesis involves progressive synaptic degeneration, a process potentially driven by maladaptive microglial pruning activity. While synaptic loss is a hallmark of AD, the molecular signals triggering pathological microglia-mediated synaptic engulfment remain elusive. Clec7a—a key marker of disease-associated microglia (DAM)—is known to activate spleen tyrosine kinase (SYK) signaling, enhancing Aβ phagocytosis and neuroprotective functions in 5×FAD models. However, its role in regulating synapse–microglia interactions under tauopathic conditions remains undefined. Our analysis revealed a progressive activation of the Clec7a–SYK signaling axis in the hippocampus of PS19 tauopathy mice, correlating with disease progression. Spatial mapping demonstrated a significant co-localization of Clec7a with hippocampal microglia, suggesting cell-autonomous signaling. The pharmacological inhibition of Clec7a achieved multimodal therapeutic effects by attenuating microglial hyperreactivity, suppressing neuroinflammatory cytokine release, and restoring physiological synaptic turnover. Mechanistically, we identified MD2 as a synaptic “eat-me” signal on tauopathy-related synapses, recruiting Clec7a+ microglia to drive aberrant synaptic elimination in PS19 mice. Strikingly, Clec7a blockade rescued hippocampal-dependent memory deficits in behavioral tests. These findings position Clec7a as a context-dependent therapeutic target, with inhibition strategies showing particular promise for tauopathy-related synaptic degeneration.

## 1. Introduction

Alzheimer’s disease (AD) is a progressive neurodegenerative disorder characterized by the accumulation of amyloid-β (Aβ) plaques and hyperphosphorylated tau tangles within the brain, accompanied by neuroinflammation, synaptic loss, and neuronal degeneration [1]. Synaptic dysfunction is a hallmark pathological feature of AD and is widely regarded as the principal driver of cognitive decline.

Microglia are the resident immune cells of the central nervous system (CNS). They play a pivotal role in preserving neuronal integrity by modulating neurotransmitter release and facilitating neuronal repair and regeneration. The continuous formation and remodeling of synapses are essential processes that ensure the proper functioning of neural circuits and the regulation of neurotransmitter dynamics. During brain development, microglia remove surplus or poorly functioning synapses via phagocytosis, ensuring the establishment of healthy neural circuits [2]. However, in pathological conditions such as AD, microglia may prune synapses abnormally, leading to synaptic damage, neural network dysfunction, and ultimately, neuronal loss and cognitive decline [3,4,5].

Tau pathology is closely linked to microglia activation and synaptic loss. Studies have shown that tau protein accumulates in the synaptic terminals of neurons, which actively secrete full-length or fragmented tau proteins [6,7]. Extracellular tau can activate microglia in many ways. For example, pre-formed tau fiber (PFF) activates microglia through the TLR2/MyD88 pathway, which leads to microglia inflammation [8]. In addition, extracellular tau can also trigger the exposure of phosphatidylserine on the surface of neurons, which increases the phagocytic activity of microglia [9]. The PS19 mouse model is a P301S mutant expressing the human microtubule-associated protein tau, which shows the pathological characteristics of tau related to AD. The activation of microglia usually occurs at the age of 3–4 months in PS19 mice [10]. In tau mice, tau pathology not only upregulates the TFEB–V-ATPase signaling pathway and regulates lysosomal homeostasis in microglia [11], but also activates the cGAS–IFN signaling pathway in microglia [12]. In addition, tau pathology inhibits the AMPK activity of neurons and promotes the lipid droplet accumulation and abnormal activation of microglia [13]. Taken together, these findings suggest that the diffusion of tau pathology is an early event that can trigger the activation of microglia.

As a key receptor involved in the microglial phagocytosis process, Clec7a is specifically expressed in myeloid-monocytic lineage cells, including microglia [14]. The receptor is characterized by several functional domains: an extracellular C-type lectin domain, a transmembrane region, and an intracellular tyrosine activation motif (ITAM). Upon ligand binding, the ITAM recruits spleen tyrosine kinase (SYK), triggering its phosphorylation, subsequently activating NF-κB to initiate the transcription of pro-inflammatory genes [15]. Clec7a has been shown to regulate phagocytosis by macrophages in multiple peripheral tissues. Moreover, Clec7a is upregulated in microglia during neurodegeneration and is an important receptor for microglial activation in response to AD pathology. Furthermore, Clec7a signals through SYK to enhance the phagocytosis of Aβ, thereby contributing to the clearance of amyloid deposits associated with AD [16]. Although the function of Clec7a mainly focuses on the clearance of pathological substances, its over-activation may lead to inflammation and synaptic damage. An in-depth study on the dual mechanism of Clec7a in AD will provide important targets and strategies for AD treatment.

In this study, we discovered that Clec7a is specifically upregulated in the microglia of the ventral hippocampus (vHPC) of 6-month-old PS19 mice. This increase in Clec7a is positively correlated with microglial activation. Through the Clec7a–MD2 signaling pathway, synapse loss is mediated. Importantly, blocking Clec7a attenuates microglial synaptic phagocytosis and neuroinflammation, thereby improving cognitive and memory dysfunction. This finding provides a new target for the early diagnosis of AD and offers a novel approach for its treatment.

## 2. Results

### 2.1. Synapse Loss and Microglia Activation in the Hippocampus of PS19 Mice

**Figure 1:**
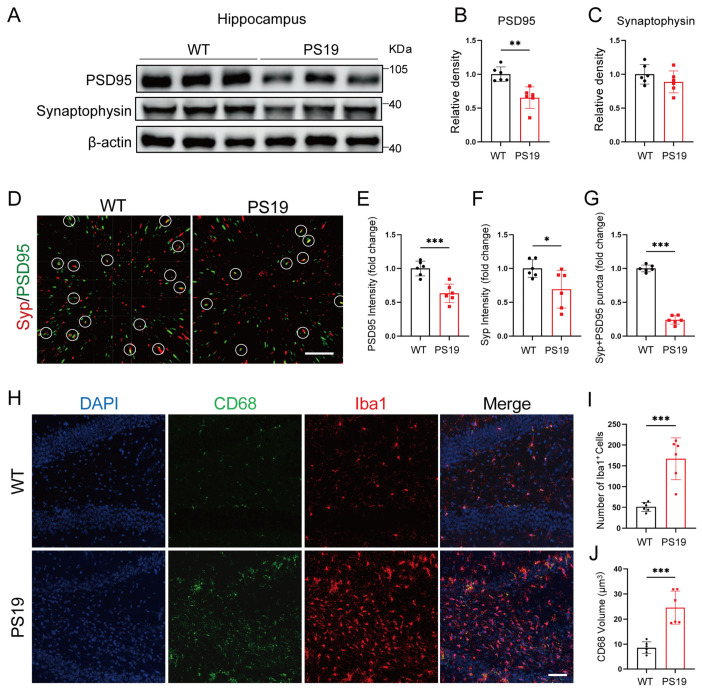
Synapse loss and microglia activation in the hippocampus of PS19 mice. (**A**) Synaptic proteins extracted from the hippocampus of 6-month-old wild-type (WT) and PS19 mice were analyzed via Western blotting. (**B**,**C**) Quantitative analysis of PSD95 (**B**) and synaptophysin (**C**) expression levels (*n* = 6 mice per group, unpaired Student’s *t*-test). (**D**) Representative single-plane images depicting the localization of PSD95 (green) and synaptophysin (Syp) (red) in the hippocampus of 6-month-old WT and PS19 mice. Dotted circles highlight colocalized synapses. Scale bars, 5 μm. (**E**–**G**) Quantification of synaptic density (*n* = 6 mice per group, unpaired Student’s *t*-test). (**H**) Immunofluorescence images showing CD68+ lysosome content (green) within Iba1^+^ microglia (red) in the hippocampus of 6-month-old WT and PS19 mice. Scale bars, 50 μm. (**I**) Quantification of the number of Iba1^+^ microglial cells (*n* = 6 mice per group, unpaired Student’s *t*-test). (**J**) Quantification of CD68+ lysosome volume (*n* = 6 mice per group, unpaired Student’s *t*-test). * *p* < 0.05; ** *p* < 0.01; *** *p* < 0.001. Data are presented as mean ± SD.

To explore microglia-mediated synapse elimination in the context of tau pathology, we used the PS19 mouse model. We first evaluated the expression levels of PSD95 and synaptophysin in both the hippocampus (Figure 1A) and cortex (Figure S1A) of PS19 mice and their wild-type (WT) littermates. Our findings showed a significant reduction in PSD95 expression in the hippocampus of PS19 mice compared to their WT counterparts (Figure 1B) and there was a trend toward a reduced expression of synaptophysin (Figure 1C). However, no noticeable differences were found in the protein levels of PSD95 and synaptophysin between the cortex of PS19 and WT mice (Figure S1B,C). Confocal imaging of Synaptophysin+ (Syp) pre-synapses and PSD95+ post-synapses (Figure 1D) confirmed a notable decrease in synaptic density in PS19 mice (Figure 1E–G). Furthermore, we evaluated microglial activation by performing immunofluorescence staining for the microglial marker Iba-1 and the macrophage/microglia lysosomal marker CD68 in the hippocampus (Figure 1H). Consistent with previous studies [10], we observed a marked increase in the number of Iba1^+^ cells and the size of CD68 fluorescence puncta in PS19 mice (Figure 1I,J), indicating that tau pathology enhances the activation of microglia.

### 2.2. Clec7a Is Specifically Increased in the Hippocampus of PS19 Mice and Closely Associated with Synaptic Loss

**Figure 2:**
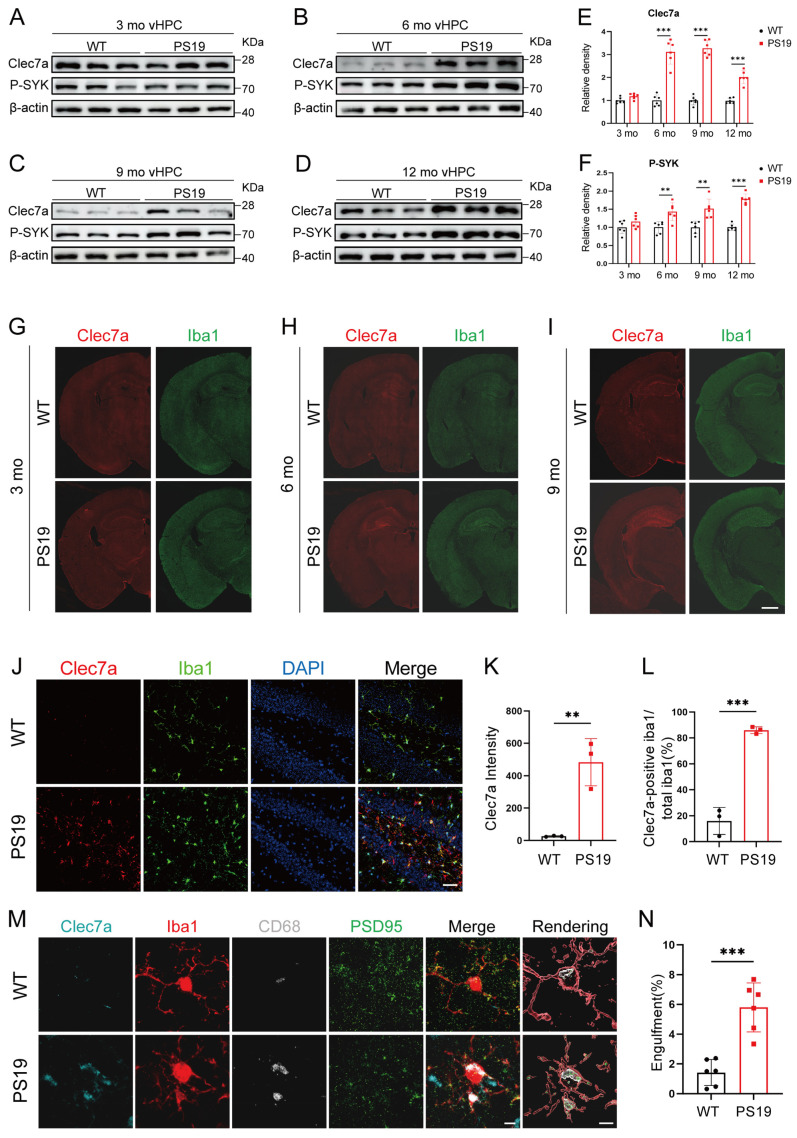
Clec7a is specifically increased in the hippocampus of PS19 mice and closely associated with synaptic loss. (**A**–**D**) Representative immunoblots of Clec7a and P-SYK in ventral hippocampal tissue from WT or PS19 mice at 3 months (**A**), 6 months (**B**), 9 months (**C**), and 12 months (**D**) of age. (**E**,**F**) Quantification of Clec7a and P-SYK levels shown in panel (**A**–**D**) (*n* = 6 mice per group, unpaired Student’s *t* test). (**G**–**I**) Representative confocal images of Clec7a (red) and Iba1^+^ microglia (green) in the whole brain of WT and PS19 mice at 3 months (**G**), 6 months (**H**), and 9 months (**I**). Scale bars, 1 mm. (**J**) Representative images showing Iba1 (green) and Clec7a (red) staining in the dentate gyrus (DG) regions of hippocampal tissue from WT and PS19 mice at 6 months. Scale bars, 50 μm. (**K**,**L**) Quantitative analysis of Clec7a intensity and the number of Clec7a-positive Iba1 (*n* = 3 mice per group, unpaired Student’s *t* test). (**M**) Confocal images of synaptic puncta engulfed by microglia showing the colocalization of PSD95 with CD68 within microglia in the DG regions of hippocampal tissue from WT and PS19 mice at 6 months. Scale bars, 5 μm. (**N**) Quantitative analysis of (**M**) (*n* = 6 mice per group, unpaired Student’s *t* test). ** *p* < 0.01; *** *p* < 0.001. Data are presented as mean ± SD.

A subset of microglia in β-amyloid–laden brain displays a disease-associated microglia (DAM) phenotype, characterized by elevated Clec7a expression [17,18]. To explore the role of Clec7a in the PS19 mouse model of tauopathy, we assessed the expression of Clec7a and phosphorylated SYK (p-SYK) in the ventral hippocampus (vHPC) (Figure 2A–D), dorsal hippocampus (dHPC) (Figure S1D–G), and cortex (Figure S2A–D) of PS19 mice and their wild-type (WT) littermates at 3, 6, 9, and 12 months. Our results revealed no significant differences in Clec7a protein expression between WT and PS19 mice in these regions at 3 months. However, Clec7a and p-SYK levels in the vHPC of PS19 mice at 6 months were markedly elevated compared to WT controls, with further increases observed at 9 and 12 months (Figure 2E,F), while the Clec7a and p-SYK expression in the dHPC or cortex of PS19 mice was increased starting from 9 months or later (Figures S1H,I and S2E,F). Taken together, these findings indicate that the Clec7a–SYK signaling pathway is progressively activated in the hippocampus of PS19 mice.

To further explore the relationship between microglial activation and Clec7a expression, we performed immunofluorescence imaging of PS19 mouse brains using antibodies against Clec7a and Iba1. We observed age-dependent activation of microglia. At 3 months, Iba1 staining was faint throughout the brain, but by 6 months, a substantial increase in Iba1 staining was specifically observed in the dentate gyrus (DG) region of PS19 mice. By 9 months of age, the proliferation of microglia spread to the whole hippocampus. In contrast, there was no age-dependent change in the microglia of WT mice at the ages of 3, 6, and 9 months (Figure 2G–I). Significantly, the distribution and density of Clec7a corresponded closely to that of the microglia. Compared with WT mice, the Clec7a expression increased significantly in the DG region of PS19 mice at 6 months, and its expression in the whole hippocampus region of PS19 mice at 9 months was markedly elevated (Figure 2G–I). Further investigation revealed a significant increase in both Clec7a intensity and the number of Clec7a-positive Iba1 cells in the dentate gyrus (DG) region of 6-month-old PS19 mice (Figure 2J–L). We then assessed the extent of PSD95-immunoreactive synaptic puncta that were internalized into Clec7a-positive microglial lysosomes in the DG region of both WT and PS19 mice at 6 months. The results indicated a marked increase in the phagocytosis of synapses by Clec7a-positive microglia in PS19 mice (Figure 2M,N), suggesting that Clec7a might play a role in synapse loss in PS19 mice.

### 2.3. Inhibition of Clec7a Effectively Mitigates Microglial Synaptic Phagocytosis and Neuroinflammation in PS19 Mice

**Figure 3:**
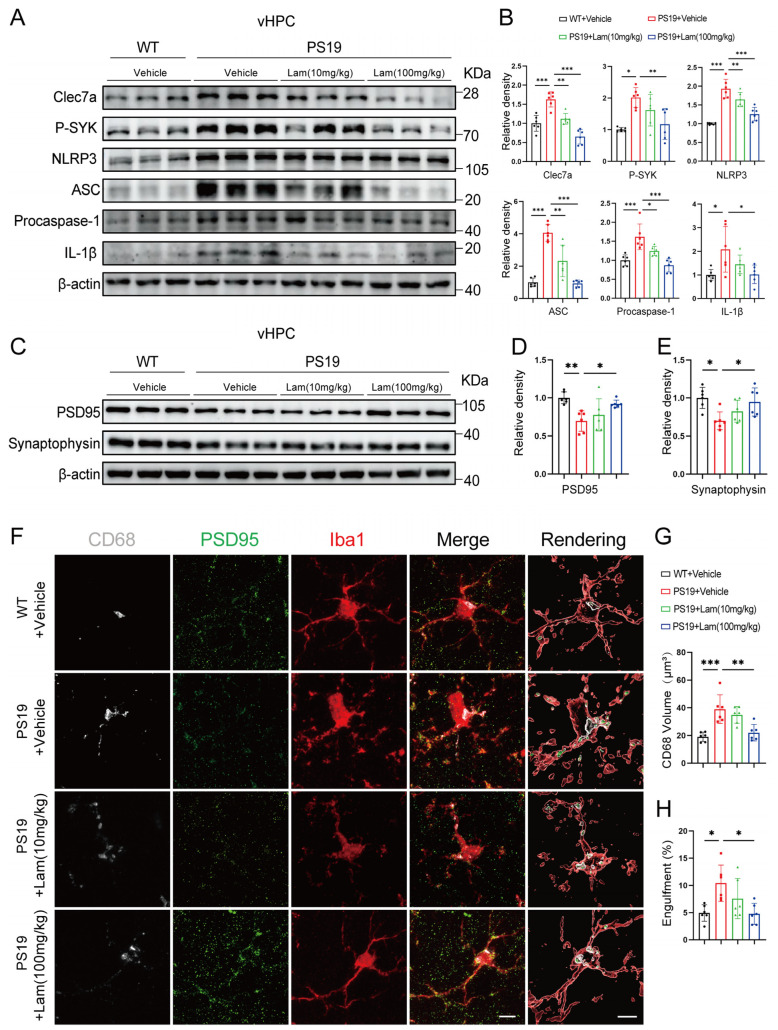
Inhibition of Clec7a effectively mitigates microglial synaptic phagocytosis and neuroinflammation in PS19 mice. (**A**) Representative Western blots of indicated proteins in the ventral hippocampal lysates from 6-month-old WT mice and Laminarin-administered PS19 mice. (**B**) Quantitation of Clec7a, P-SYK, NLRP3, ASC, Procaspase-1, and IL-1β proteins in (**A**) (*n* = 6 mice per group, unpaired Student’s *t* test). (**C**) Representative Western blots of indicated proteins in the ventral hippocampal lysates from 6-months old WT mice and Laminarin-administered PS19 mice. (**D**,**E**) Quantitation of PSD95 (**D**) and synaptophysin (**E**) proteins (*n* = 6 mice per group, unpaired Student’s *t* test). (**F**) Confocal images of synaptic puncta engulfed by microglia showing the colocalization of PSD95 (green) with CD68 (grays) within Iba1^+^ microglia (red) in the DG regions of hippocampal tissue from WT mice and Laminarin-administered PS19 mice. Scale bars, 5 μm. (**G**) Quantitation of CD68 volume. (**H**) Quantitation of PSD95 engulfment within CD68+ microglial structures (*n* = 6 mice per group, one-way ANOVA test). * *p* < 0.05; ** *p* < 0.01; *** *p* < 0.001. Data are presented as mean ± SD.

It has been found that in 5×FAD mice, the inhibition of SYK suppresses the activation of microglial NLRP3 inflammasomes, which reduces IL-1β and IL-18 production [19]. In a mouse model of myocardial infarction, small interfering RNA silencing of Clec7a downregulates the expression of NLRP3, IL-1β, and IL-18, thereby attenuating myocardial injury [20]. These studies suggest that Clec7a may be closely related to NLRP3-mediated neuroinflammatory responses. To investigate whether Clec7a regulates microglial inflammatory responses through the SYK–NLRP3 pathway in PS19 model mice, Laminarin (Lam), an inhibitor of Clec7a, was administered by intraperitoneal injection. Remarkably, Laminarin significantly decreased the levels of Clec7a and p-SYK proteins in the hippocampus of 6-month-old PS19 mice, indicating the suppression of the Clec7a–SYK pathway activation (Figure 3A,B and Figure S3A–D). Furthermore, protein levels of NLRP3, ASC, cleaved caspase-1, and IL-1β were notably increased in the PS19 vehicle group compared to the WT vehicle group. In contrast, the expression of these proteins was significantly reduced in PS19 mice that received Laminarin treatment (Figure 3A,B and Figure S4A–D). These findings indicate that the blockage of Clec7a substantially attenuates neuroinflammation in the hippocampus of PS19 mice.

Building on our observation that Clec7a correlates with the microglial engulfment of synapses in PS19 mice, we hypothesized that the inhibition of Clec7a might promote synaptic recovery in these mice. To test this, we assessed the effect of the Clec7a inhibitor Laminarin on synapse. Immunoblotting results revealed that Laminarin treatment significantly increased the levels of PSD95, with a concomitant trend toward elevated synaptophysin expression (Figure 3C–E). Remarkably, Laminarin treatment not only significantly decreased the overall Iba1^+^ and CD68+ signals but also decreased PSD95 puncta in CD68+ microglial structures in the PS19 mice (Figure 3F–H), demonstrating that the blockage of Clec7a restricts the microglial engulfment of synapses.

### 2.4. Blockage of Clec7a Improves Cognitive and Memory Deficits in PS19 Mice

**Figure 4:**
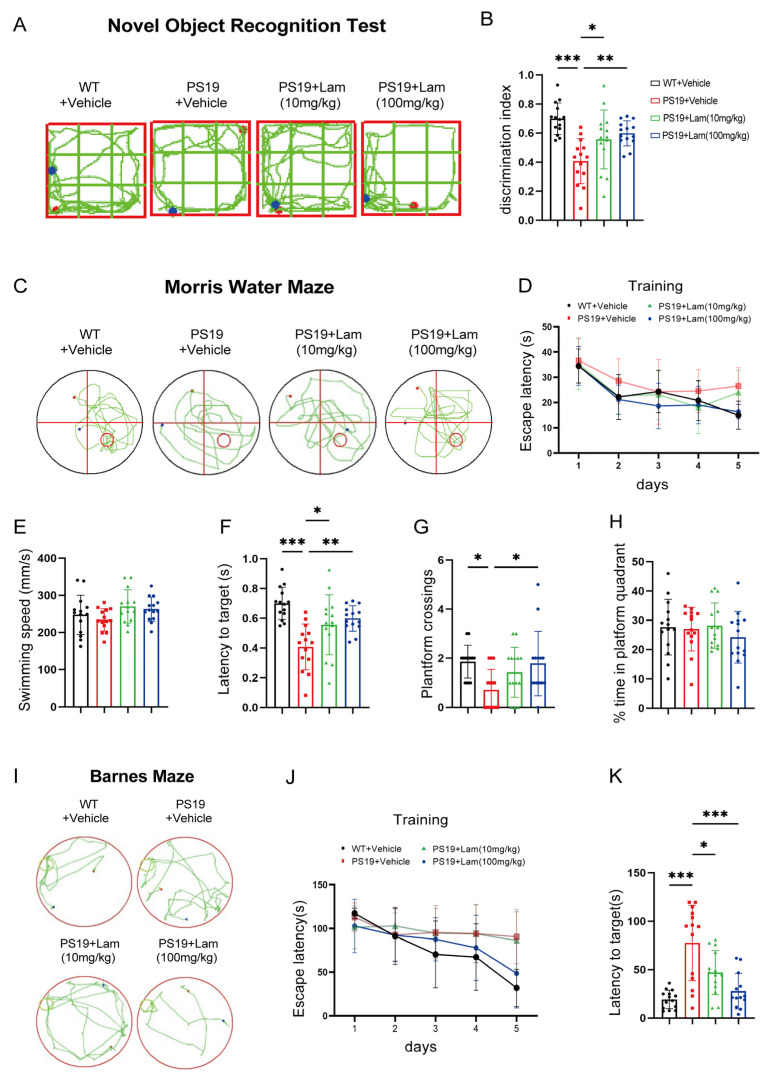
Blockage of Clec7a improves cognitive and memory dysfunction in PS19 mice. PS19 and WT littermates (5–6 months old) were administered 10 or 100 mg/kg/day Laminarian or vehicle for 2 weeks and then tested. (**A**) The novel object recognition (NOR) task was used to assess recognition memory in PS19 mice after intraperitoneal administration with the Laminarian. Exploration time spent with the familiar and novel objects was measured for each mouse (**B**) Discrimination index in the NOR task (*n* = 14 mice per group, one-way ANOVA test). (**C**) Representative navigation trajectories of mice in the morris water maze (MWM). (**D**) The average escape latencies for the five learning trials. (**E**) The swimming speed. (**F**) Latency to mount the submerged platform. (**G**) Number of entries onto the platform. (**H**) Percentage of time in platform quadrant. (**I**) Representative trajectory images showing the movement of mice in the Barnes maze during the probe phase. (**J**) Latencies in the Barnes maze for the five learning trials. (**K**) Primary latencies to the target hole in the Barnes Maze task. *n* = 13–14 mice per group, one-way ANOVA test. * *p* < 0.05; ** *p* < 0.01; *** *p* < 0.001. Data are presented as mean ± SD.

To evaluate the cognitive consequences of Clec7a inhibition, we conducted comprehensive behavioral assessments in PS19 tauopathy mice. The novel object recognition test revealed a marked decrease in novelty preference in PS19 mice treated with physiological saline, with a discrimination index (DI) of 41%, compared to 70% in age-matched wild-type (WT) controls, indicating memory impairment. In contrast, PS19 mice treated with Laminarian (100 mg/kg) showed an improved DI of 60%, spending more time with the novel object, suggesting the recovery of memory function (Figure 4A,B).

The Morris water maze (MWM) test, consisting of both training and testing phases, is commonly employed to assess cognitive function in mice. During the training phase, the PS19 + vehicle group exhibited significantly longer escape latencies on days 4 and 5 compared to the WT + vehicle group (Figure 4C,D), indicating cognitive impairments in the PS19 + vehicle group. On day 5, interestingly, the PS19 + Laminarian (100 mg/kg) group demonstrated a significant reduction in escape latencies compared to the PS19 + vehicle group (Figure 4D), suggesting that the inhibition of Clec7a improves cognitive function in PS19 mice. After the five-day training period, the platform was removed to evaluate the swimming speed and the number of platform crossings across the four groups. Swimming speeds were similar among all groups, indicating that motor functions were unaffected (Figure 4E). Additionally, the PS19 + vehicle group exhibited significantly fewer platform crossings and longer escape latencies than the WT + vehicle group (Figure 4F,G), indicating impaired cognitive function. Importantly, PS19 + Laminarian (100 mg/kg) mice demonstrated significantly more platform crossings and shorter escape latencies than PS19 + vehicle mice (Figure 4F,G), further suggesting that blocking Clec7a can ameliorate cognitive deficits in PS19 mice, although there were no significant differences in time spent in the platform quadrant among the four groups (Figure 4H). Consistently, treatment with the Laminarin also restored the spatial memory function of PS19 mice in the Barnes Maze task (Figure 4I–K). Altogether, our data suggest that the suppression of Clec7a signals is sufficient to restore cognitive and memory deficits.

### 2.5. Clec7a–MD2 Signaling Mediates Synaptic Loss in PS19 Mice

MD2, also known as lymphocyte antigen 96, is an important molecule closely associated with immune responses. It primarily recognizes and responds to bacterial lipopolysaccharides (LPS) through its interaction with TLR4, thereby initiating the body’s immune response. However, recent studies suggest that MD2 may also be involved in microglia-mediated synapse elimination after ischemic stroke as a microglia Clec7a ligand [21]. Through immunofluorescent microscopy, MD2 immunoreactivity was predominantly immunoreactive for NeuN, a general neuron marker, and a small amount of MD2 is also punctuate in the extra-neuronal space as well as in microglia. None of the MD2 overlapped with markers of astrocytes [glial fibrillary acidic protein (GFAP)] (Figure S5A–C). We established cultures of primary neurons, microglia, and astrocytes. Using Western blot analysis, we confirmed that MD2 is predominantly produced by neurons, whereas microglia and astrocytes generate only minimal amounts of MD2 (Figure S6A,B). Additionally, we quantified MD2 levels in the culture media of these three cell types. MD2 was detected in the supernatant of primary neuron cultures, suggesting that ex-tracellular MD2 is primarily secreted by neurons (Figure S6C).

**Figure 5:**
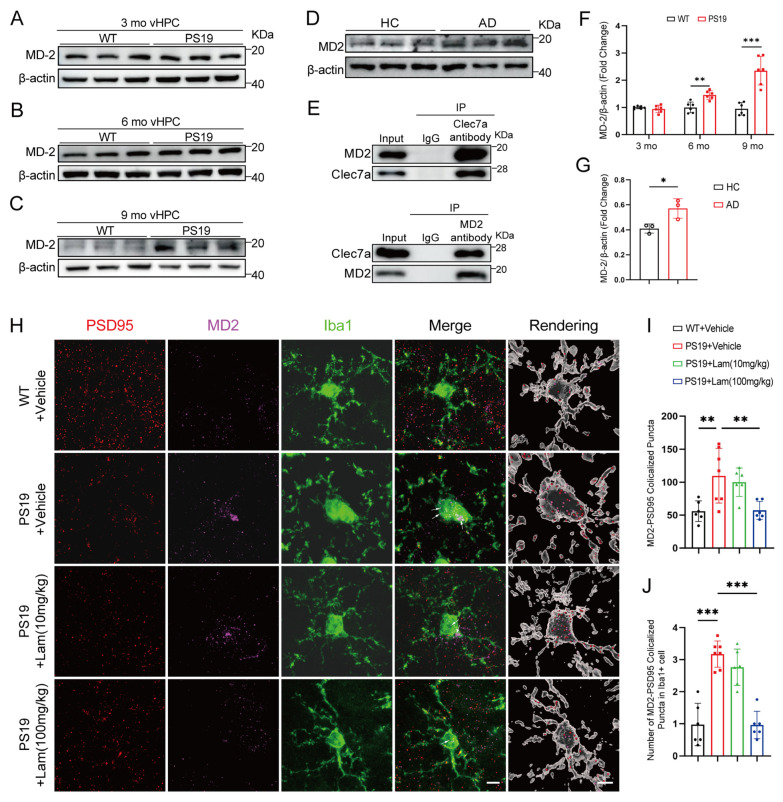
Clec7a–MD2 signaling mediates synaptic loss in PS19 mice. (**A**–**C**) Representative immunoblots of MD2 in ventral hippocampal lysate from WT or PS19 mice at 3 months (**A**), 6 months (**B**), and 9 months (**C**). (**D**) Representative immunoblots of hippocampus samples from AD patients. (**E**) Endogenous Clec7a was co-immunoprecipitated with MD2. (**F**) Quantitation of MD2 levels shown in panel (**A**–**C**) (*n* = 6 mice per group, unpaired Student’s *t* test). (**G**) Quantitation of MD2 amounts in (**D**) (*n* = 3 biologically independent samples per group, unpaired Student’s *t* test). (**H**) High resolution confocal images and 3D reconstructions show the presence of MD2 (magenta) tagged PSD95 (red) within Iba1^+^ cells (green) in the DG regions of hippocampal tissue from Laminarin-administered PS19 mice. White arrows indicating PSD95+ MD2+ puncta in Iba1^+^ cell. Scale bars, 5 μm. (**I**) Quantitation of MD2-PSD95 colocalized puncta (*n* = 6 mice per group, one-way ANOVA test). (**J**) Quantitation of MD2-PSD95 colocalized puncta in Iba1^+^ cell (*n* = 6 mice per group, one-way ANOVA test). * *p* < 0.05; ** *p* < 0.01; *** *p* < 0.001. Data are presented as mean ± SD.

We analyzed the expression levels of MD2 in the hippocampus and cortex of PS19 mice and their WT littermates at 3, 6 and 9 months of age. No significant differences in the hippocampal MD2 were observed between WT and PS19 mice at 3 months (Figure 5A,F and Figure S7A,D). However, a marked increase was detected in the vHPC and dHPC of PS19 mice at 6 months (Figure 5B,F and Figure S7B,D), with a further elevation at 9 months (Figure 5C,F and Figure S7C,D). In contrast, no significant differences were observed in MD2 protein levels in the cortex of PS19 and WT mice (Figure S7E–H). These results indicate that the expression of MD2 in PS19 mice is consistent with that of Clec7a in time and space. We further examined the protein amounts of MD2 in post-mortem AD patients and healthy control hippocampal lysates (Figure 5D). The results showed that the expression of MD2 in the hippocampus of AD patients increased (Figure 5G). Co-immunoprecipitation (CoIP) assays revealed that endogenous Clec7a interacts with MD2 (Figure 5E).

To gain a deeper understanding of the roles of Clec7a and MD2 in the microglial phagocytosis of synaptosomes, mouse brain slices from Figure 3 were double labeled with antibodies against MD2 and PSD95. Immunofluorescence analysis revealed that the colocalization of MD2 with PSD95 puncta were much more remarkable in the DG region of PS19 + vehicle mice compared to WT + vehicle mice and the colocalization was reduced by the administration of Laminarian (Figure 5H,I). To further examine the MD2-labeled synaptic puncta in microglia, we conducted a 3D reconstruction analysis. We observed that compared with WT + vehicle mice, microglia in the DG region of PS19 + vehicle mice engulfed more MD2-labeled PSD95-positive synapses. Notably, Laminarian treatment in PS19 mice resulted in fewer MD2-labeled, PSD95-positive synapses within microglia, suggesting that blocking Clec7a reduces the microglial engulfment of MD2-labeled synapses (Figure 5H,J). Taken together, these results indicate that Clec7a –MD2 signaling mediates synaptic loss in PS19 mice.

## 3. Discussion

Microglia-mediated synaptic phagocytosis is emerging as a crucial mechanism in the pathophysiology of various neurological disorders, including autism [22], schizophrenia [23,24], depression [25], and AD [26,27]. Previous studies have demonstrated that aberrant microglial activation and excessive synaptic pruning are implicated in cognitive decline, synaptic dysfunction, and neuronal loss [28,29,30]. However, the specific molecular pathways that drive microglial-mediated synaptic elimination remain poorly understood. Our study offers novel insights into the role of Clec7a in modulating microglial synaptic phagocytosis in the context of tau pathology, highlighting its potential as a therapeutic target in AD.

Clec7a, a C-type lectin receptor expressed by microglia, has recently been identified as a key player in microglial activation and synaptic pruning in the response to AD pathology [17,18]. Moreover, the knockdown of microglial Clec7a significantly impairs the microglial-mediated phagocytosis of synapses [21]. In 5×FAD mice, CLEC7A-induced activation of SYK promotes the microglial phagocytosis of Aβ [16]. Consistent with findings in 5×FAD mice, our study indicates that Clec7a is significantly upregulated in the vHPC microglia of PS19 mice, a tauopathy model (Figure 2G–I). This upregulation correlates with increased microglial activation and synaptic loss, reinforcing the hypothesis that Clec7a-mediated phagocytosis is involved in the progression of tau-induced neurodegeneration (Figure 1). Moreover, we demonstrate that the activation of the Clec7a–SYK signaling pathway is spatially and temporally regulated in the hippocampus, particularly as the disease progresses in PS19 mice (Figure 2A–F). Notably, our study expands the current understanding of Clec7a’s role by examining its dynamic expression across different stages of tau pathology. Unlike previous studies that primarily focused on Clec7a’s role in amyloid pathology, our work provides a more comprehensive analysis of its involvement in tauopathy. The observed age-dependent increase in Clec7a expression suggests that it may serve as an early marker of disease progression, as microglial activation and synaptic phagocytosis are both enhanced at the 6-month stage in PS19 mice (Figure 2A–I). This supports the notion that Clec7a activation is closely tied to the exacerbation of tau-related pathologies in the brain. Additionally, our immunofluorescence analyses showed that Clec7a expression closely mirrors the distribution of microglia, suggesting a functional relationship between these two factors (Figure 2J–N).

Our study also highlights the potential therapeutic application of Clec7a inhibition. Previous studies have shown that inhibiting Clec7a using Laminarin, a non-toxic polysaccharide, can reduce microglial-mediated synaptic phagocytosis in various models [31]. In this study, we reveal that the pharmacological inhibition of Clec7a effectively mitigates tauopathy-associated synaptic degeneration and neuroinflammatory responses in the PS19 tauopathy mouse model (Figure 3 and Figure 4). Mechanistic investigations demonstrate that the administration of the selective Clec7a inhibitor Laminarin produces dual regulatory effects by: (1) significantly downregulating Clec7a expression and suppressing SYK phosphorylation (p-SYK), thereby effectively blocking the Clec7a signaling cascade and reducing the expression levels of NLRP3 inflammasome components; and (2) concomitantly attenuating microglia-mediated synaptic engulfment. Our integrated findings establish a critical regulatory role of Clec7a in coordinating microglial neuroinflammatory responses through two parallel mechanisms: cytokine-mediated inflammatory signaling and direct synaptic phagocytic activity. Importantly, pharmacological intervention targeting Clec7a achieves therapeutic efficacy by normalizing microglial activation states, suppressing neurotoxic cytokine production, and restoring physiological synaptic maintenance patterns. This multimodal modulation ultimately rescues hippocampal-dependent cognitive functions, as evidenced by significant improvements in spatial memory performance. These results position Clec7a as a promising therapeutic target for tauopathies including Alzheimer’s disease. Interestingly, the role of Clec7a in neurodegenerative diseases appears context dependent. While Clec7a activation has been shown to exacerbate neuroinflammation in ischemic stroke models [32], it promotes phagocytosis and synaptic loss in AD models [33,34]. This dichotomy underscores the complex and multifaceted role of Clec7a in the immune response within the central nervous system. The variability in its role across different diseases suggests that targeting Clec7a could be a delicate balance, requiring careful consideration of disease context and stage.

Moreover, microglial-mediated synaptic phagocytosis is tightly regulated by complex molecular signals that enable microglia to recognize target sites and trigger phagocytosis. For example, C1q and C3 are found at developing synapses, where they facilitate the microglial engulfment of synaptic inputs through the CR3 receptor [35,36]. These results suggest that immune signaling pathways in the CNS play a role in the regulation of synaptic pruning. As established in earlier studies, Clec7a acts as a receptor that facilitates the microglial phagocytosis of synapses. Furthermore, it has been demonstrated that MD2 could potentially serve as a novel ligand for microglial Clec7a [21]. Our study identifies critical ligand–receptor interactions that orchestrate microglia-mediated synaptic phagocytosis. Specifically, we reveal that MD2 serves as a novel endogenous ligand for Clec7a, establishing a phagocytic signaling axis that directs Clec7a-expressing microglia to MD2-labeled synaptic compartments and promotes their elimination (Figure 5). This mechanistic insight into the MD2–Clec7a signaling pathway not only advances our understanding of microglial synaptic pruning mechanisms but also presents a promising therapeutic target for modulating neuroinflammatory responses in neurodegenerative disorders. Further investigation of this ligand-receptor interaction and its downstream signaling cascades may yield novel intervention strategies for diseases characterized by aberrant synaptic remodeling.

This study has several limitations. We observed increased MD2 expression in the hippocampus, direct contact between MD2 and synapses, and the enrichment of synaptic material within microglia in PS19 mice. However, these findings do not directly confirm that MD2 functions as an “eat me” signal for phagocytosis. Specific interventions targeting MD2 are needed to further elucidate its role in synapse loss. Additionally, our results indicate that under normal physiological conditions, MD2 is predominantly expressed in neurons, with minimal expression in microglia and astrocytes. In contrast, MD2 expression was detected in microglia in PS19 mice, likely resulting from the phagocytosis of MD2-positive synaptic material by these cells. Furthermore, previous studies have shown that astrocytes can also remove synapses in disease contexts [37,38]. This suggests that astrocytes might similarly engulf MD2-positive synapses, a possibility that warrants further investigation in future experiments.

In conclusion, our study elucidates the role of Clec7a in microglial synaptic phagocytosis in tau pathology, offering compelling evidence that targeting Clec7a could mitigate synaptic loss and neuroinflammation in tauopathies. These findings provide a foundation for the development of Clec7a-targeted therapies aimed at slowing the progression of Alzheimer’s disease and other neurodegenerative disorders characterized by synaptic dysfunction.

## 4. Materials and Methods

### 4.1. Mice

C57 and PS19 mice (7 weeks old, weighing 23 ± 3 g) were purchased from Beijing Vitalstar Biotechnology Co., Ltd. (Beijing, China). The mice were housed in a controlled specific pathogen-free (SPF) environment, with continuous access to food and water. At the commencement of the study, all mice were 24 weeks old. Animal handling procedures adhered to the International Guidelines for the Use of Laboratory Animals and received approval from the Medical Ethics Committee of Jianghan University. Molecular experiments were carried out on both WT and PS19 mice at ages 4, 6, 9, and 12 months. Previous research has indicated that cognitive deficits appear in PS19 mice as early as 4 months. For the current study, 5-month-old WT and PS19 mice were selected for intraperitoneal injections.

### 4.2. Human Sample Tissue

Brain tissue samples were procured from the Chinese Brain Bank Center at Zhongnan University of Science and Technology, comprising six samples from three patients diagnosed with Alzheimer’s disease (1 male, 2 females; aged 85 ± 10 years) and three control subjects (1 male, 2 females; aged 85 ± 10 years). All brain samples were collected with written consent from the respective families of the deceased individuals. This study was conducted in accordance with ethical guidelines and received approval from the Medical Ethics Committee of Jiang Han University (approval JHDXLL2022-043).

### 4.3. Drug Administration

The Clec7a antagonist Laminarian (L9634, Sigma-Aldrich, St. Louis, MO, USA) was diluted to a concentration of 10 mg/mL in saline, according to the manufacturer’s guidelines. For intraperitoneal injection, 5-month-old PS19 mice were administered either Laminarian or an equal volume of saline daily for two weeks at doses of 10 mg/kg or 100 mg/kg. Following the behavioral assessments, the mice received one final injection before being sacrificed. Brain tissue was subsequently collected for biochemical analysis or immunofluorescence staining.

### 4.4. Culture of Primary Neurons, Astrocytes and Microglia

Primary neurons, astrocytes, and microglia were isolated from the hippocampi of C57BL/6 mouse pups. Briefly, hippocampal tissues were dissected, minced, and digested in a digestion buffer containing 0.25% trypsin and 0.01% EDTA in 1× PBS for 15 min at 37 °C in a 5% CO_2_ incubator. The digestion was terminated by adding 10% FBS-DMEM/F12 medium, followed by filtration through a 70 μm cell strainer. The filtrate was centrifuged at 1200 rpm for 10 min at 4 °C, and the supernatant was discarded. The resulting cell pellet was resuspended in 10% FBS-DMEM/F12 medium and passed through a mesh filter. The neuronal suspension was diluted appropriately for cell counting and plated in six-well plates at a density of 7 × 10^5^ cells per well, with each well containing 2 mL of culture medium. The plates were gently shaken to ensure even cell distribution and incubated under 5% CO_2_ at 37 °C. After 4–6 h, the 10% FBS-DMEM/F12 medium was replaced with maintenance medium (97% Neurobasal, 2% B27, 1% GlutaMAX, and 1% Penicillin-Streptomycin) for continued culture.

For primary astrocyte isolation, the cell pellet obtained from the previous centrifugation step was resuspended in 10% FBS-DMEM/F12 medium and seeded into 25 cm^2^ (T25) culture flasks. The mixed hippocampal cells were maintained at 37 °C with 5% CO_2_, and after 24 h, the medium was replaced to remove floating debris. Thereafter, the culture medium was changed every three days. After 7–10 days, the flasks were placed on an orbital shaker (250 rpm, 37 °C) for 16 h to detach microglia. The supernatant containing the detached microglia was removed, and the remaining confluent astrocyte layer was collected and transferred to 75 cm^2^ (T75) culture flasks for further expansion. The medium was replaced every three days, and after two weeks, astrocytes were digested with digestion buffer, collected, and used for subsequent experiments.

For primary microglia isolation, the mixed hippocampal cultures were maintained for 3–4 weeks, with medium changes every five days. Following this period, the supernatant was discarded, and the remaining adherent layer was washed with 1× D-Hanks solution. To detach microglia, the cells were incubated with 12 mmol/L lidocaine hydrochloride (diluted in 1× D-Hanks) at 37 °C for 20 min. The flasks were then gently tapped to dislodge microglia growing on the astrocyte layer. The detached microglia were collected from the supernatant and centrifuged at 3000 rpm for 10 min at 4 °C. The purified primary microglia were resuspended in culture medium and used for subsequent experiments.

### 4.5. Western Blotting

Tissue samples were washed with pre-chilled PBS and lysed on ice in RIPA buffer with protease inhibitors for 30 min. After centrifugation at 13,000 rpm for 20 min at 4 °C, the supernatant was collected and protein concentration was adjusted to 5 mg/mL. Proteins were separated by electrophoresis and transferred to an NC membrane. The membrane was blocked with 5% non-fat milk for 1 h, and incubated with primary antibody overnight at 4 °C, followed by secondary antibody incubation and visualization. The following antibodies were used: anti-β-actin (GB15001-100, Servicebio, Wuhan, China); anti-Clec7a (AF1756-SP, R&D, Minneapolis, MN, USA); anti-Clec7a (YN2088, Immunoway, Wuhan China); anti-MD2 (11784-1-AP, Proteintech, Wuhan, China); anti-mouse IL-1β (AF-401-NA, R&D); anti-mouse Caspase-1 (24232, Proteintech); anti-NLRP3 (15101, Proteintech); anti-Mouse IgG(H + L) (A00001, Zenbio, Chengdu, China); anti-PSD95 (A0131, ABclonal, Wuhan, China); and anti-Synaptophysin (A19122, ABclonal)

### 4.6. Immunofluorescence and Microscopy

Mice were perfused with saline solution and then 4% PFA, the brains were then transferred to a 10–30% sucrose solution for 3 days before embedding for cryosectioning. Frozen sections, 30 µm thick, were blocked at room temperature for 1 h in 3% normal goat serum and 0.1% Triton-X 100, followed by overnight incubation with the primary antibodies at 4 °C. The primary antibodies used in this study included Clec7a (1:200, AF1756-SP, R&D, Minneapolis, MN, USA); MD2(1:200, GTX85517, GeneTex, San Antonio, TX, USA); Iba-1 (1:1000, ab178846, Abcam, Cambridge, MA, USA); Iba-1 (1:500, ab283346, Abcam, Cambridge, MA, USA); NueN (1:500, 66836-1-lg, Proteintech, Wuhan, China); GFAP (1:500, 3670, CST, Danvers, MA, USA); synaptophysin (1:200, ab16659, abcam, Cambridge, MA, USA); PSD95 (1:200, MAB1596, Millipore Sigma, Burlington, MA, USA); and CD68 (1:200, MCA1957, Bio-RAD, Inc., Hercules, CA, USA), followed by incubation with secondary antibodies (1:1000, CST, Danvers, MA, USA).

### 4.7. Behavioral Assessments

#### 4.7.1. Novel Object Recognition

The experiment was performed in a rectangular arena (40 × 40 cm), with the floor cleaned using 70% ethanol between each trial to remove any scent cues. Prior to the experiment, mice were allowed to explore the empty arena for 10 min to habituate to the environment. During the training phase, two identical objects were placed in opposite corners of the arena. Mice were introduced to the arena and permitted to explore the objects for 10 min. The total time spent exploring each object was recorded. Twenty-four hours later, one of the familiar objects was replaced with a novel object, while the other object remained unchanged. The mouse was given 10 min to explore, and the time spent with both the familiar and novel objects was recorded for analysis.

#### 4.7.2. Morris Water Maze

Mice were trained in a circular pool (120 cm in diameter, 50 cm in height) filled with water maintained at 22 ± 1 °C and rendered opaque with non-toxic white paint. The pool was divided into four quadrants, with a hidden platform (10 cm in diameter) submerged 1 cm below the water’s surface in the target quadrant. Over the course of 5 days, mice underwent four trials each day. In each trial, lasting 60 s, mice were placed at random starting positions and given the opportunity to locate the hidden platform. If the platform was not found within the time limit, mice were guided to it and allowed to remain there for 10 s. Following the training phase, the platform was removed, and mice were allowed to swim freely for 60 s. The time spent in the target quadrant and the number of platform crossings were then recorded for analysis.

#### 4.7.3. Barnes Maze

The behavioral test consisted of five days of training and one day of testing. The experimental device was a disk with a diameter of 90 cm. There are 22 holes evenly distributed at 5 cm along the edge of the disk, of which only one hole is equipped with an escape box. A bright light source was placed above the platform, forcing the mice to explore and hide in the box. During the training period of 5 consecutive days, the mice were trained once a day. If the mice did not enter the escape box within 300 s, the mice were guided into it, and the mice were removed and put back into the cage 30 s later. The tests were conducted three days apart after the training phase. During the test period, each mouse was active for 120 s. Software was used to record data in real time during behavioral processes. Finally, the time when the mice entered the escape box was noted.

### 4.8. Co-Immunoprecipitation (Co-IP)

For the immunoprecipitation of MD2, 1.5 μg of biotinylated rabbit anti-MD2 antibody (GTX85517, GeneTex) was incubated with 400–500 μg of protein sample overnight at 4 °C. For the immunoprecipitation of Clec7a, 1.5 μg of biotinylated goat anti-Clec7a antibody (AF1756-SP, R&D) was incubated with the protein sample. The antibody-antigen complexes were captured using Protein G magnetic beads (HY-K0204, MCE, Monmouth Junction, NJ, USA) and incubated at 4 °C for 2 h. To eliminate non-specific proteins, the beads were washed four times with IP lysis buffer. Following this, the beads were boiled in SDS-PAGE loading buffer and analyzed by immunoblotting. The blots were probed with rabbit anti-Clec7a (1:500, mabg-mdct, Invivogen, San Diego, CA, USA) and mouse anti-MD2 (1:500, 11784-1-AP, Proteintech, Wuhan, China) antibodies.

### 4.9. Statistical Analysis

Data are presented as means ± standard deviations (SDs) from three or more independent experiments and were analyzed using GraphPad Prism (version 8.0). A one-way analysis of variance (ANOVA) was employed to assess significant main effects and group differences among three or more groups, with post-hoc analysis conducted using Tukey’s or least significant difference (LSD) multiple comparisons. For time-course studies, a two-way ANOVA with Bonferroni corrections was used to evaluate significance. Between-group ratios were analyzed using the Kruskal–Wallis test, followed by Dunn’s multiple comparisons. A *p*-value of <0.05 was considered statistically significant.

## Data Availability

The data that support the findings of this study are available from the corresponding author upon reasonable request.

## Supplementary Materials

The following supporting information can be downloaded at: https://www.mdpi.com/article/10.3390/ijms26072888/s1.
