# Supplementary material for: Clec7a Signaling in Microglia Promotes Synapse Loss Associated with Tauopathy
Source: Int J Mol Sci. 2025 Mar 22;26(7):2888. doi: 10.3390/ijms26072888 (PMC11988799; doi:10.3390/ijms26072888)
Supplement: Supplementary file 1 [file ijms-26-02888-s001.zip › ijms-3517918-supplementary.pdf]

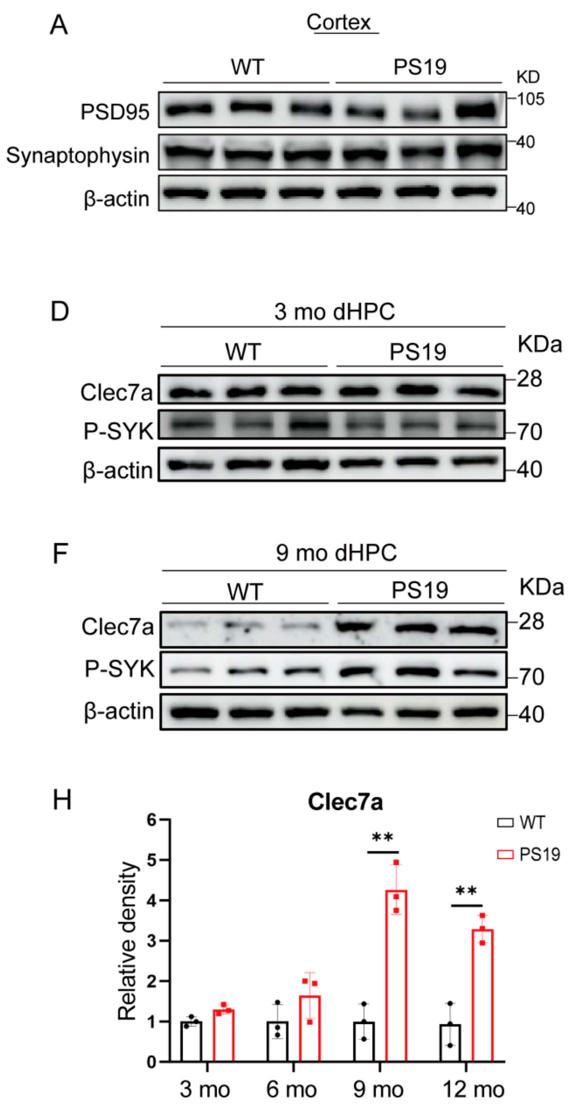

Figure S1. Clec7a and P-SYK level is increased in the dHPC of PS19 mice. (**A**) Synaptic proteins from the cortex of 6-months old WT and PS19 mice were analyzed by western blotting. (**B-C**) Quantitation analysis of PSD95 (B) and synaptophysin (C) (n = 3 mice per group, unpaired Student's t test). (**D-G**) Representative immunoblots of Clec7a and P-SYK in dorsal hippocampal lysate from WT or PS19 mice at 3 months (D), 6 months (E), 9 months (F), and 12 months (G) of age. (**H-I**) Quantification of Clec7a (H) and P-SYK levels (I) (n = 3 mice per group, unpaired Student's t test). *p < 0.05; **p < 0.01; ns, not significant. Data are presented as mean ± SD.

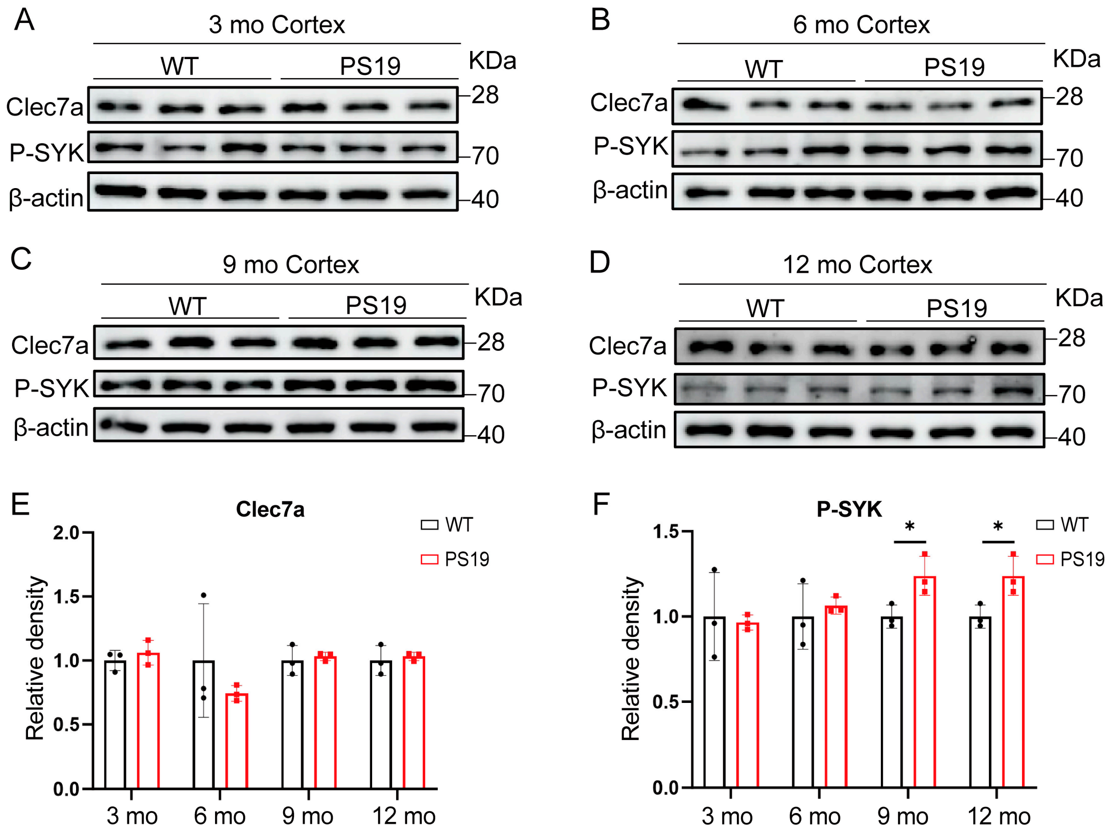

Figure S2. Clec7a level is not increased in the cortex of PS19 mice. (**A-D**) Representative immunoblots of Clec7a and P-SYK in cortex lysate from WT or PS19 mice at 3 months (A), 6 months (B), 9 months (C), and 12 months (D) of age. (**E-F**) Quantification of Clec7a (E) and P-SYK (F) levels (n = 3 mice per group, unpaired Student's t test). *$p < 0.05$; ns, not significant. Data are presented as mean ± SD.

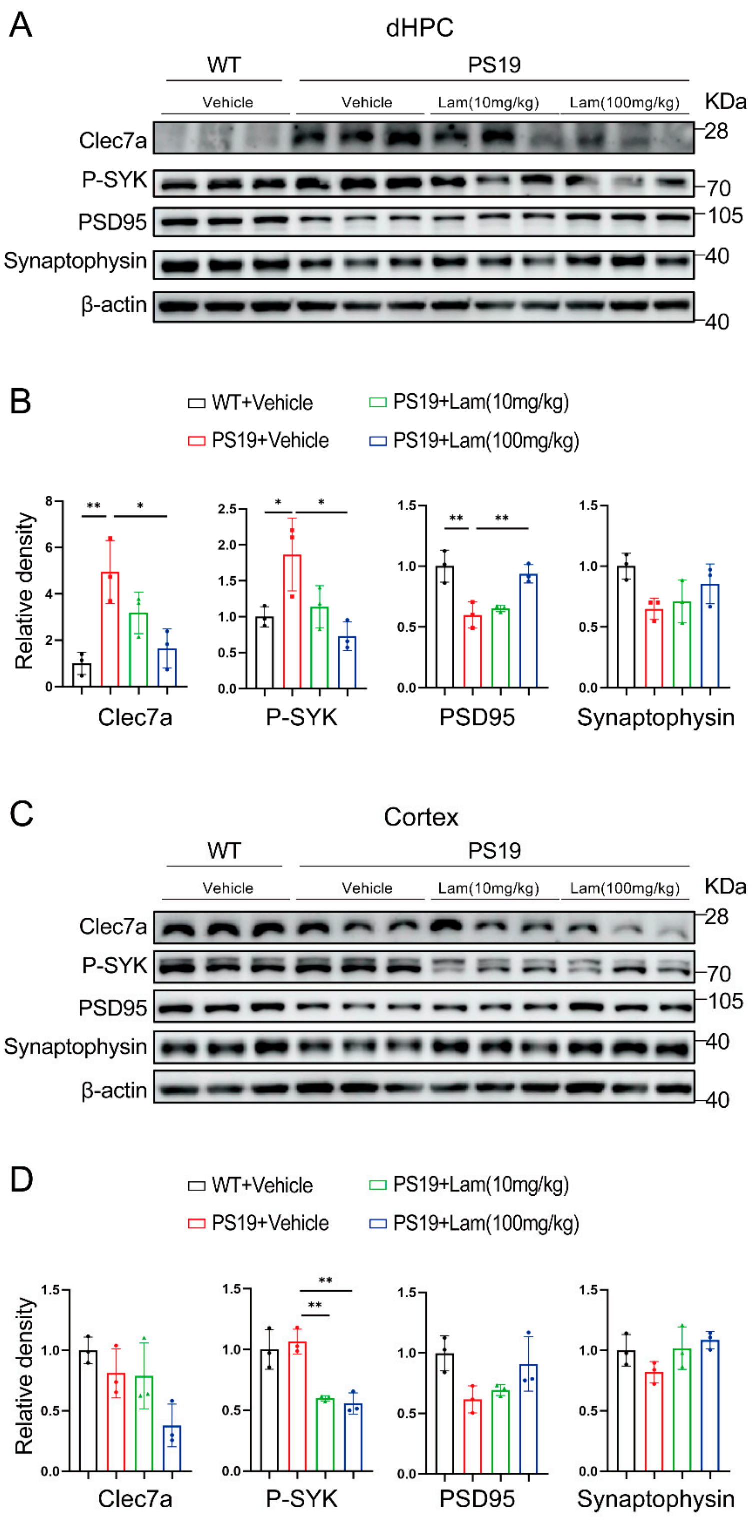

Figure S3. Laminarin ameliorates synaptic protein loss in both the dHPC and cortex of PS19 mice. (**A**)Representative western blots of indicated proteins in the dorsal hippocampal lysates from Laminarin-administered 6-months old PS19 mice. (**B**)Quantitation of Clec7a, P-SYK, PSD95 and Synaptophysin proteins in (A) (n = 3 mice per group, unpaired Student's t test). (**C**)Representative western blots of indicated proteins in the cortex lysates from Laminarin-administered PS19 mice. (**D**)Quantitation of Clec7a, P-SYK, PSD95 and Synaptophysin proteins in (C) (n = 3 mice per group, unpaired Student's t test). *p < 0.05; **p < 0.01; ***p < 0.001; ns, not significant. Data are presented as mean ± SD.

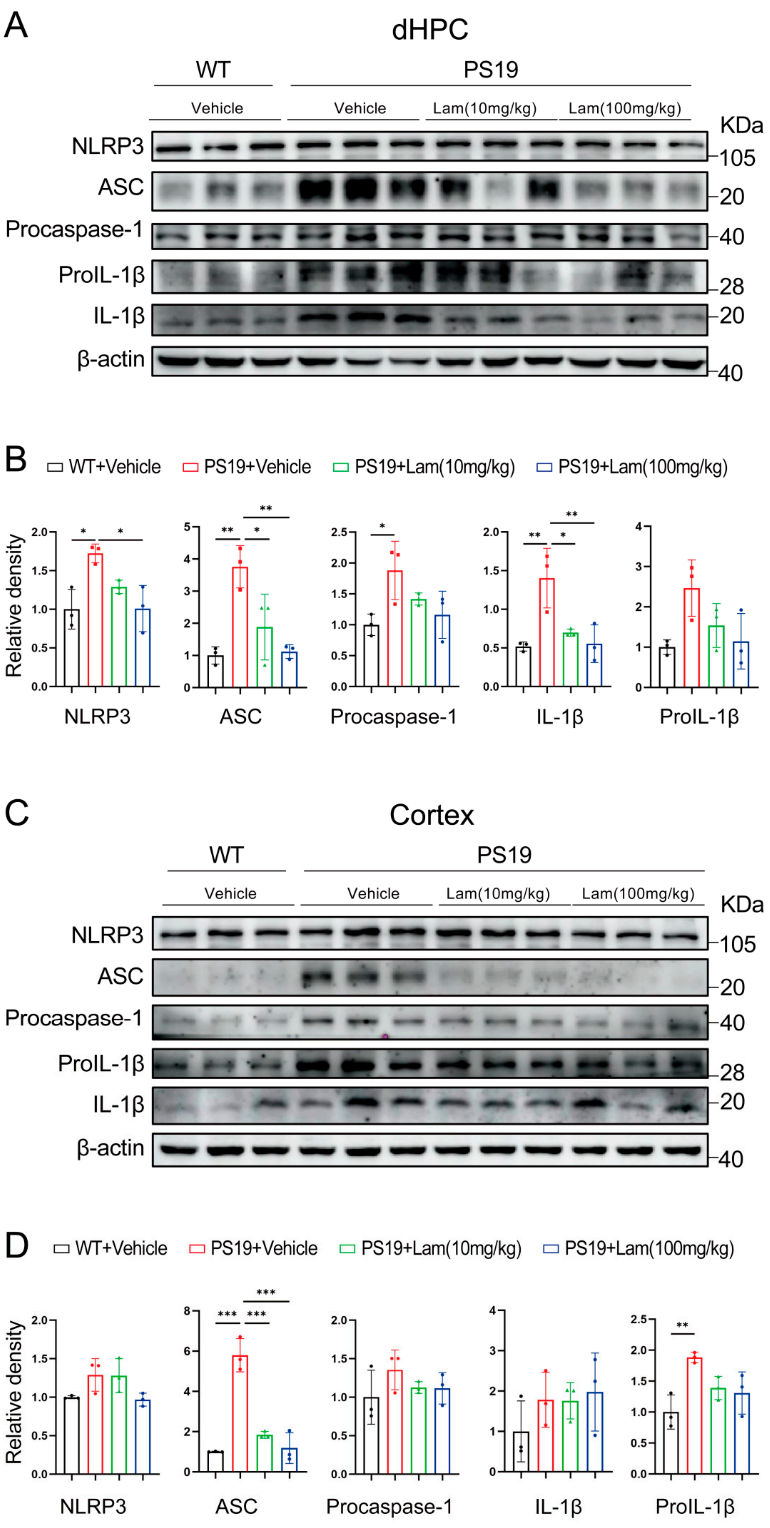

Figure S4. Laminarin improved the neuroinflammation in the dHPC and cortex of PS19 mice. (**A**)Representative western blots of indicated proteins in the dorsal hippocampal lysates from Laminarin-administered 6-months old PS19 mice. (**B**)Quantitation of NLRP3, ASC, Procaspase-1, ProIL-1β and IL-1β proteins in (A) (n = 3 mice per group, unpaired Student's t test). (**C**)Representative western blots of indicated proteins in the cortex lysates from Laminarin-administered PS19 mice. (**D**)Quantitation of NLRP3, ASC, Procaspase-1, ProIL-1β and IL-1β proteins in (C) (n = 3 mice per group, unpaired Student's t test). $^*p < 0.05$; $^{**}p < 0.01$; $^{***}p < 0.001$; ns, not significant. Data are presented as mean ± SD.

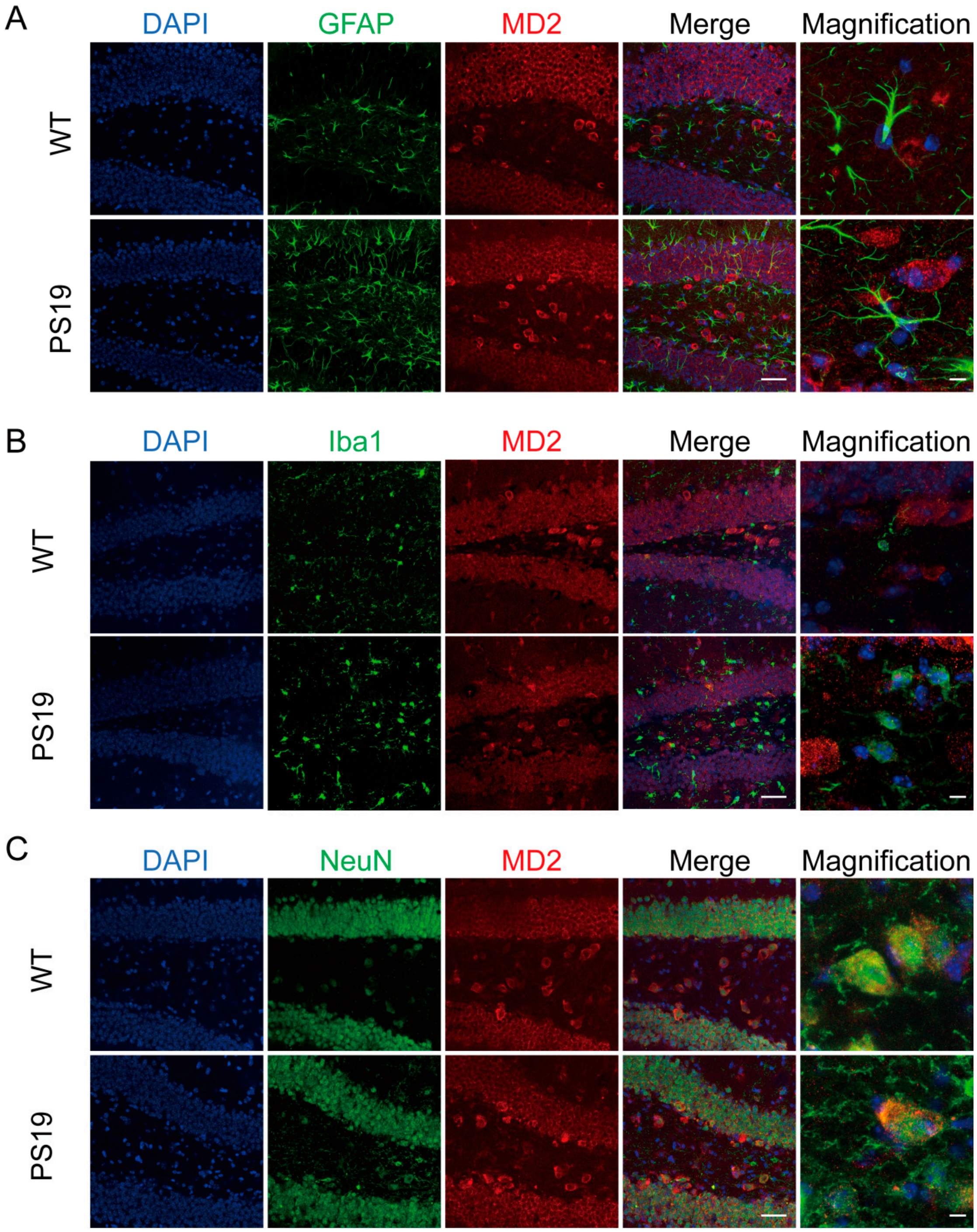

Figure S5. MD2 is predominantly localized in neurons. (**A**) Representative immunostaining of $MD2^+$ (red) and $GFAP^+$ (green) cells in the DG regions of hippocampal tissue from WT and PS19 mice at 6 months. (**B**) Representative immunostaining of $MD2^+$ (red) and $Iba1^+$ (green) cells in the DG regions of hippocampal tissue from WT and PS19 mice at 6 months. (**C**) Representative immunostaining of $MD2^+$ (red) and $NeuN^+$ (green) cells in the DG regions of hippocampal tissue from WT and PS19 mice at 6 months.

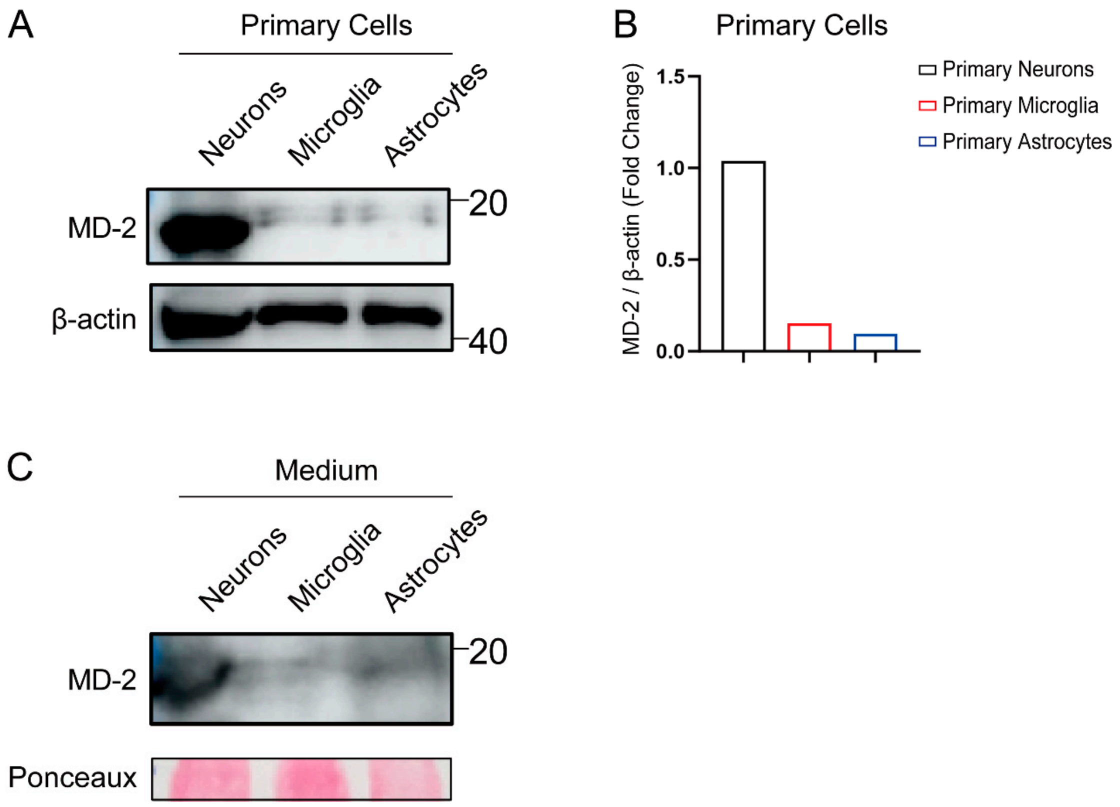

Figure S6. MD2 is mainly produced and secreted by neurons. (**A-B**) Representative Western blot analysis and quantification of MD-2 in primary neurons, primary astrocytes, and primary microglia. (**C**) The culture supernatant of primary neurons，primary microglia and primary astrocytes cells were collected and subjected for analysis of MD2 expression by western blot.

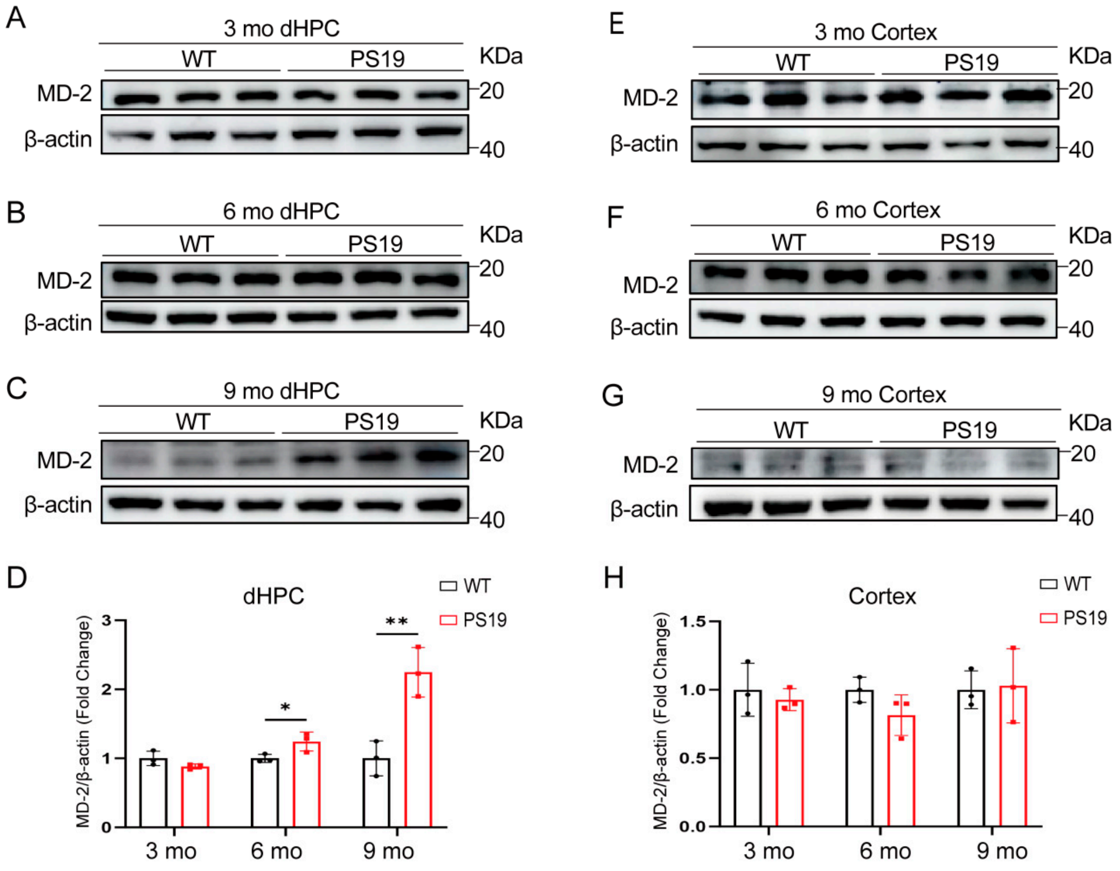

Figure S7. MD2 increased in an age-dependent manner in the dHPC of PS19 mice. (**A-C**) Representative immunoblots of MD2 in dorsal hippocampal and cortex lysate from WT or PS19 mice at 3 months (A), 6 months (B), 9 months (C). (**D**) Quantitation of MD2 levels shown in panel (A-C) (n = 3 mice per group, unpaired Student's t test). (**E-G**) Representative immunoblots of MD2 in cortex lysate from WT or PS19 mice at 3 months. (E), 6 months (F), 9 months (G). (**H**) Quantitation of MD2 levels shown in panel (E-G) (n = 3 mice per group, unpaired Student's t test). *$p < 0.05$; **$p < 0.011$; ns, not significant. Data are presented as mean ± SD.
